# Longterm outcome of anal fistula – A retrospective study

**DOI:** 10.1038/s41598-020-63541-3

**Published:** 2020-04-16

**Authors:** Christos Andreou, Jasmin Zeindler, Daniel Oertli, Heidi Misteli

**Affiliations:** 1 Clarunis, University Center for Gastrointestinal and Liver Diseases, Basel, Switzerland; 2 Clinique de Genolier, Genolier, Switzerland; 3 Department of abdominal Surgery, Spital Uster, Zürich, Switzerland

**Keywords:** Anus, Chronic inflammation, Digestive signs and symptoms

## Abstract

This retrospective observational study analyses the outcomes of patients undergoing surgery for anal fistula at a single centre in order to assess recurrence and re-operation rates after different surgical techniques. During January 2005 and May 2013, all patients with anal fistula were included. Baseline characteristics, details of presentation, fistula anatomy, type of surgery, post-surgical outcomes and follow-up data were collected. The primary endpoints were long-term closure rate and recurrence rate after 2 years. Secondary endpoints were persistent pain, postoperative complications and continence status. A total of 65 patients were included. From a total amount of 93 operations, 65 were fistulotomies, 13 mucosal advancement flaps, 7 anal fistula plugs and 8 cutting-setons. The mean follow up was 80 months. Healing was achieved in 85%. The highest recurrence rate was seen in anal fistula plug with 42%. On the other hand, no recurrence was observed in the cutting-seton procedures. For all included operation no persistent postoperative pain nor incontinence was observed. In conclusion, despite all existing anal fistula operations up to date, the optimal technique with low recurrence rate and assured safety for the anal sphincter is still lacking. Nonetheless, according to our promising results for the cutting-seton technique, this technique, otherwise considered obsolete, should be further evaluated in a prospective study.

## Introduction

The annual incidence of anal fistula varies from 10–30/100.000 with a sex ratio of 2–3 men per woman. The average age of onset is between 20 and 50 years^1^.

Although in the 4^th^ century BC, Hippocrates addressed the clinical relevance of anal fistula, the first that proposed a classification for this surgical illness was Parker and colleagues in 1976^2^. The Park’s classification system deals with anal fistulae that have cryptoglandular infection and separates them into four categories depending on the relation to the sphincter muscle: intersphincteric, transsphincteric, suprasphincteric, and extrasphincteric. A simpler classification used nowadays is describing anal fistula in relation to the dentate line. Anal fistulae originated below the dentate line are therefore classified as low and those originated above as high. One important purpose of classification is for decision making of the possible surgical treatment for anal fistula in order to evaluate the risk of recurrence and faecal incontinence after surgical treatment in the long term. However, the treatment of anal fistula remains challenging. The goals of treatment are draining infection, eradicating the fistula and avoiding persistent or recurrent disease while preserving the anal sphincter function.

The globally accepted and gold standard surgical treatment for low anal fistula is the technique of fistulotomy. For this surgical procedure, the fistula can either be divided with leaving the fistula tract open to epithelialize, or a fistulectomy with complete excision of the fistula tract can be performed. Compared with the fistulotomy, the fistulectomy is slightly more demanding. Especially because of the inflammatory changes on the fistula walls, more damage can be caused to the tissues surrounding the fistula during the fistulectomy. There is no strong evidence showing a clear advantage of one technique over the other regarding the healing rate. In 1999 Belmonte Montes *et al*. compared the two techniques in a randomized controlled trial, showing that in fistulectomy there is a higher risk for sphincter lesions compared to fistulotomy^3^. However, Ratto *et al*. stated in their review, comparing fistulotomy alone with fistulectomy and primary sphincteroplasty, lower incontinence rate for the more demanding procedure^4^. The fistulotomy operating time is slightly shorter compared to fistulectomy, but statistically significant^5^. The success rate after fistulotomy ranges from 80–100%^6–10^ with a rate of faecal incontinence from 0–62%^9,11^.

For higher and more complex fistulae sphincter-sparing operations are recommended as more ideal approaches, for preserving faecal continence. The most well-established technique is the mucosal advancement flap operation. This technique was originally developed to treat rectovaginal fistula and has been transposed for treating high anal fistula. The principle of this operation is to obliterate the primary internal orifice by interposing a flap of rectal mucosa. In theory, the technique of mucosal advancement flap is preserving the sphincter muscle and should therefore not result in increased incontinence postoperatively. According to the literature the long term closure rate is around 70–98%^12–14^. However, the creation of the flap harbours the risk of muscle damage, which is mirrored in some studies showing impaired continence in 10% - 21% of patients up to 45% in patients with suprasphincteric fistulae or Crohn’s disease^15^. In recent years, in the effort to maximize the safety of the patient and lower the risk of postoperative incontinence after treating high transphincteric fistulae, several new approaches for the surgical treatment of high anal fistulae have emerged.

One of those approaches targeting high transphincteric fistulae is the anal fistula plug. The plug is a natural biomaterial harvested from porcine small intestine and fabricated into a biomedical product of various shapes and thickness. The plug is introduced into the fistula tract after cleaning the tract. The purpose of this sphincter-sparing technique is to put a scaffold material into the fistula tract for the patient’s fibroblasts to migrate and promote wound healing of the tract. The big advantage of this operation is that it is an easy procedure with low morbidity and incontinence rate. However the healing rate after this type of operation is not very promising, showing a healing rate between 20%–60%^14,16–18^. Failure with this technique usually occurs within the first month following the intervention, mainly due to early expulsion of the plug.

A further possible and long known technique is the placement of a cutting-seton. Hippocrates was the first to use this technique where he inserted horsehair with lint in the fistula tract and then periodically tightened it. It is used almost for all types of anal fistulae and is showing a high healing rate between 80–100%^19,20^*.* However there is a lot of controversy regarding this type of operation and generally, nowadays it is not anymore recommended for anal fistula treatment because of a relevant incontinence rate in some studies with numbers fluctuating between 0–63%^10,21,22^.

Apart from the before mentioned operations, many more techniques are described in the literature such as the ligation of intersphincteric fistula tract (LIFT)^23^, the treatment with expanded adipose-derived stem cells (ASC)^24^, fistula laser closure (FiLaC)^25^ and video-assisted anal fistula treatment (VAAFT)^26^. The diversity of operations underlines the complexity of the disease and is further making evident that until now there is no simple surgical technique with an optimal outcome for the patient.

In this retrospective observational study, we analyzed all operatively treated fistulae in our department. With having results about recurrence rate and reoperation rate after each surgical technique we plan to establish treatment recommendations in order to improve the outcome of anal fistula treatment in our hospital in the future.

## Results

The total amount of analyzed operations was 93 (100%) in a total of 79 fistulae as seen in Table 1. All operations for anal fistula were performed in a total of 65 patients, 80% males (n = 52) and 20% being females (n = 13) with a mean age of 49.7 years [SD ± 15.9]. The different techniques of the performed operations consisted of 65 fistulotomies (70%), 13 mucosal advancement flap (14%), 7 anal fistula plug (8%) and 8 cutting-seton operations (8%). The mean follow up period was 80 months (defined as the time beginning after the patient’s first operation) and healing was achieved in 85%. The total rate of recurrences was 15% (n = 14).

**Table 1:** Results.

	Number of Operations (%)	Recurrence (%)	Complications (%)	Incontinence
Total	93 (100)	14 (15)	7 (8)	0
Fistulotomy	65 (70)	8 (12)	7 (11)	0
Mucosa Advancement Flap	13 (14)	3 (23)	0	0
Anal Fistula Plug	7 (8)	3 (42)	0	0
Cutting Seton	8 (8)	0	0	0

In a total of 13 mucosal advancement flap operations, there were 3 recurrences with a recurrence rate of 23% in a mean follow up period of 80.2 months. In a total of 7 anal fistula plug operations there were 3 recurrences with a recurrence rate of 42%. In 65 fistulotomy operations, 8 recurrences occurred with a total recurrence rate of 12%. Because of this unexpected high recurrence rate after fistulotomy operations all these operation reports were revised in detail. The analysis showed, when separating high from low fistulae, that 18 of the 65 fistulotomy operations were performed in high fistulae. As shown in Table 2, five of the 8 recurrences after fistulotomy, occurred after fistulotomy in high fistulae whereas only 3 recurrences occurred after fistulotomy in low fistulae. Signifying for the patients treated with fistulotomy, a 28% recurrence rate was observed in the high fistula group and a 6% recurrence rate in the low fistula group. After looking further into the treatment algorithm of those high fistulae and further shown into Table 3, it could be seen that 4 (80%) of the 5 recurrences in high fistulae were not treated with a seton drain before the final operation.

**Table 2:** Fistulotomy operation in high fistulae ± Seton drain.

Fistulotomy	Number of Operations (%)	Recurrence (%)
Overall	18 (100)	5 (28)
+ Seton drain	7 (40)	1 (14)
- Seton drain	11 (60)	4 (36)

**Table 3:** Seton conditioning in high fistulae.

Fistulotomy in high Fistulae	Number of Operations	Recurrence (%)
Overall	18	5 (28)
+ Seton drain	7	1 (14)
- Seton drain	11	4 (36)

In a total of 8 cutting-seton procedures, no recurrence was observed, no postoperative pain and no faecal incontinence. Looking again further into detail of the cutting-seton procedures, 6 (75%) of the fistulae treated with cutting-seton were high transsphincteric fistulae. Of the remaining 2 (25%), one was a submucosal fistula and the other was not specified. Postoperative infection in form of an abscess formation without further progression into a fistula was seen in 7 of the 65 fistulotomy procedures (Clavien-Dindo IIIb), giving a complication rate of 11% for the fistulotomy group. In all other techniques, no postoperative infection or other complications were observed.

In all 93 operations (100%) carried out with different surgical techniques, there was no difference in continence status between the patients with a mean Wexner Score of 0. Furthermore, there was no postoperative pain nor faecal incontinence neither for gas nor fluids in the long-term follow up.

## Discussion

This long-term follow up study examined closure rate, recurrence rate and re-operation rate of anal fistula treatment in association of different surgical techniques after 2 years. The surgical techniques included were fistulotomy, mucosal advancement flap, anal fistula plug and cutting-seton in a total of 93 operations. Seventy percent of the performed operations were fistulotomies (n = 65), 14% mucosal advancement flaps (n = 13), 8% anal fistula plugs (n = 7) and 8% cutting-seton operations (n = 8). The mean follow up was 80 months (range, 2–157 months), and healing was achieved in 85% with a total recurrence rate of 15%.

In the fistulotomy group, in a total of 65 operations, there was a recurrence rate of 12% which is surprisingly high in comparison of what the literature suggests^6–8^. As mentioned above, almost one-third of the fistulotomy operations (n = 18) were performed in high fistulae with a recurrence rate in this subgroup of 28% (5/18). Furthermore, in 80% of those high fistulae which were treated with fistulotomy, there was no preoperative treatment with a seton. In the fistulotomy group with no sphincter involvement (low fistulae) there was a recurrence rate of 6%. Most of these operations were performed in the acute setting of infected anorectal fistulae at the time when there was an abscess present. Even though definite treatment of an underlying fistula in the acute setting is controversial because of increased risk of faecal incontinence, this strategy shows decreased recurrence rate, comparing with abscess-drainage alone^27^. Despite this conclusion from the meta-analysis of randomized clinical trials by Quah *et al*., we saw in our clinic that the decision for primary fistulotomy in high anal fistula without the use of seton drainage had worse outcomes regarding the recurrence rate. A reason for this high recurrence rate after fistulotomies in high fistula in our hospital could be caused either from the fact that endoanal ultrasonography was not routinely performed when an acute infected anorectal fistula was operated, thus some more complex fistulae might have been mistaken for simple, or due to the fact, that in the majority of fistulotomies in high fistulae there was no seton conditioning before the intention-to-treat operation. Even though having this unexpected high recurrence rate, no patient suffered from postoperative faecal incontinence. Due to the nature of retrospective studies in general, we couldn’t always ensure that a primary spincteroplasty was made in fistulotomies of high fistulae but its mirrored from the Wexner’s Incontinence score of 0.

In the case of the mucosal advancement flap operation, the results show a recurrence rate of 23% which is again in accordance to existing literature suggesting a wide range of recurrence rate between 2–32%^12–14,16^. However, in most of the published studies, the follow up period was less than 24 months. Only a few studies show a longer follow up period, the first of which was conducted in 2006 by Der Haagen *et al*.^7^ with a follow up period of 72 months. The cumulative recurrence rate was 22% after 12 months, 44% after 24 months, and 63% in both 48 and 72 month-periods. It was shown that the recurrence rate doubled in the second year of their follow up period and rose till the 4^th^ year. After the 4^th^ year, the authors didn’t see any further recurrences. In our series, the recurrence rate was lower if we take into consideration the mean follow up period of 80.2 months. Another study by Mitalas *et al*. which had an even longer follow up period of a median 92 months, showed that the initial healing rate was 68% after mucosal advancement flap with no significant change in the recurrence rates over time^12^.

Some groups compare the recurrence rate of different techniques of the advancement flap operation. Promising results with a recurrence rate of only 10% are described for the technique of full-thickness wall flap in comparison with the mucosal advancement flap which shows a recurrence rate of 40%. Though the faecal incontinence and soiling rates tend to be higher in the full thickness groups^28^. In our study we used both types of advancement flap techniques and we didn’t subdivide them. Nevertheless, we report a 0% faecal incontinence and soiling rate.

The results of the anal fistula plug group showed a recurrence rate of 42% and therefore lies in the same range with the existing literature^14,16–18^. There is a wide range in published data and some studies even report a significantly higher recurrence rate. For instance, Safar *et al*. reported an 86.1% recurrence rate with a mean follow up of 4 months in their retrospective study of patients treated with anal fistula plug for complex anal fistulae^29^. The authors attributed the high failure rate to postoperative infection and therefore dislodgement of the plug. In our series, no postoperative infection was documented. Another randomized control study by Ortiz *et al*. was even terminated prematurely after 1 year because of a fistula recurrence in 12 of 15 patients treated with anal fistula plug^16^. As discussed before, in our study there was not always an intraoperative ultrasound used for further assessment of the fistula and thus more complex fistulae i.e with both transsphincteric and intersphincteric courses were possibly not appropriately diagnosed, which could have helped for an optimal choice for the technique of fistula treatment. Seeing the high recurrence rate after anal fistula plug one could further question the true efficacy of this technique. If we go back to the golden rules of anal fistula treatment which are high healing rate with low incontinence rate and take into consideration that anal fistula plug is a sphincter-sparing technique used in complex fistulae, a recurrence rate of 42% could be more acceptable for a technique that is so easy to perform and its repetitive use doesn’t result in higher incontinence rates.

In the group of cutting-seton operations, in a total of 8 procedures, no recurrence was documented. Additionally, no postoperative faecal incontinence nor soiling appeared. Again, this result is rather surprising if we consider that the technique by cutting-seton is a very old method of fistula management and has been no longer in use by many proctologists because of the high faecal incontinence incidence as high as 63% reported in some studies^15,21,22^. On the contrary, our study showed a high healing rate with no incontinence. Similar conclusions to our study with a high healing rate and a low faecal incontinence rate were also shown by Soliman and colleagues where they studied the recurrence rate, healing rate and complications after cutting-seton treatment in 81 patients with transsphincteric anal fistula of cryptoglandular origin^30^. They reported a healing rate of 86% with a recurrence rate of 7% from which half of them were true recurrences and half de-novo fistulae. In our study, 75% of the cutting-seton procedures were done in transsphincteric or high fistulae where more sophisticated techniques are usually preferred, such as advancement flap operations or other more modern procedures. These techniques count as sphincter-sparing procedures, even though not always successful. Furthermore, the more modern techniques often fail to show convincing results as time goes by and longer follow-up periods are available. Additionally, some modern techniques require expensive materials, such as the use of anal fistula plug whereas the cutting-seton technique only uses a simple suture and can be tightened in an outpatient clinic without the use of further anaesthetics. In our study the postoperative continence of the patients was measured using the Wexner’s score of faecal incontinence instead of an objective examination such as anal manometry or endoanal ultrasonography. Non-weighted instruments with simple numerical totals for severity, such as Wexner’s score, are highly subjective. On the other hand, weighted instruments focus on responses, which are multiplied by a weight that reflects the severity, and a score can then be calculated. In our study, we did not have any information or objective measurement of the preoperative continence status. Furthermore, most of the operations were performed 10 years ago, at a time where specialized examinations like anorectal manometry and endoanal ultrasonography were not routinely performed at our hospital, even more in the setting of the emergency treatment of an acute inflammation or sepsis. Additionally, a significant number of patients had moved and could not be followed-up by clinical examination, leaving the follow-up by phone being the only option. Even so, we believe that Wexner’s score of faecal incontinence still reflects the overall faecal continence status of the patients and their quality of life.

In the 2^nd^ quarter of 2019, the long-waited FIAT study (Fistula-in-ano Trial) from NHS (National Health Service) was published. Jayne DG *et al*. conducted a randomised, prospective, multicentre clinical trial with 304 patients which suffered from transsphincteric anal fistula with involvement more than 33% of the sphincter complex^31^, comparing anal fistula plug versus other commonly used surgical techniques. The first group of participants was treated with anal fistula plug and the second group of participants was treated with the surgical technique that the surgeon considered appropriate. In the second arm, surgeons could choose between fistulotomy, cutting seton, advancement flap or LIFT. The primary endpoint was quality of life, using by the Faecal Incontinence Quality of Life (FIQoL) score. Healing, faecal incontinence, complications, re-operation and cost-effectiveness were also measured as secondary endpoints. It took place in the United Kingdom between May 2011 and March 2016. The results showed no differences in quality of life amongst the two groups in the 12-month follow-up. The healing rate was 54% in the fistula plug arm and 55% in the surgeon’s preference arm. The only difference seen between the two groups was that patients that were treated with fistula plug had higher rates of unexpected pain in the 6-week follow-up. Also, the treatment with anal fistula plug was more expensive and only slightly improved the quality of life of the patients. Faecal incontinence was recorded at 6 weeks, at 6 months and 12 months using the St Mark’s incontinence score. The baseline incontinence scores tended to be higher in the surgeon’s preference group than in the fistula plug group, but with similar standard deviations. Interestingly, the results after comparing incontinence scores showed no significant differences in mean incontinence score between the two groups. Regarding the faecal incontinence is noteworthy that; subgroup analyses also showed that no treatment was superior to the other. That means that the 47 patients who were treated with cutting seton didn’t show increased incontinence rates when compared to the patients treated with other techniques or with anal fistula plug.

A recent meta-analysis showed recurrence rates after surgery for anal fistula ranging from 2.5% to 57.1%^32^. In the same meta-analysis, the most important risk factors for recurrence were revealed to be high transphincteric fistula, non-detected internal opening, treating the fistula only with seton drain, the presence of a horseshoe-formed abscess, more than one fistula tracts and prior anal surgery. According to existing data, the true recurrence rate for every surgical technique becomes more evident after longer follow up periods. Thus, with shorter follow up periods we might miss the true recurrence rate of a certain technique. As discussed previously this becomes evident especially in studies of more modern methods like anal fistula plug, fibrin glue or LIFT procedures, whereas promising results are shown in the short follow-up periods and the longer the follow-up period is, the more disappointing the results become with even higher recurrence rate compared to the well-established standard techniques. For example, Ky *et al*. reported a short-term success rate of anal fistula plug of over 80% at 3 to 8 weeks, with a decline down to 55% at follow up of 6.5 months^33^. A retrospective study from Chung et. al analyzed the healing rate from different anal fistula procedures of 232 patients with high or transsphincteric fistulae. The mean follow up period was 3 months and showed that anal fistula plug and advancement flap have similar healing rates after this period^34^ whereas Christoforidis et. al reported a success rate of 63% in the advancement flap group compared to only 32% in the anal fistula plug group after a mean follow up of 56 months for the advancement flap and 14 months for the anal fistula plug^14^.

Overall, reduced recurrence rates and safeguarding of the sphincter muscles are the 2 golden rules when treating anal fistulae. Especially for transsphincteric fistulae, where the treatment is more challenging and the incontinence rates are higher, these two rules need to be taken into consideration when choosing the appropriate surgical technique.

After so many years of evolving the surgical procedures throughout all fields and in proctology, anal fistula treatment remains still a challenge for the modern surgeon. Besides the traditional procedures such as fistulotomy, cutting-seton and advancement flap procedures, many newer sphincter-sparing techniques are described which promise lower risk of incontinence even after reoperations but exhibit lower healing rates in a longer follow up time^14,17^. Therefore, especially for high fistulae, even though that the ideal surgical technique is not yet known, the surgeon must consider and inform the patient that; in the case of a recurrence, a reoperation using a sphincter-sparing technique is possible with a relatively low risk for faecal incontinence.

In conclusion, despite all existing anal fistula operations up to date, the optimal technique with low recurrence rate and assured safety for the anal sphincter is still lacking. Even for the less demanding method of fistulotomy, seton conditioning and intraoperative endoanal ultrasonography are of great importance and should be always taken into consideration when treating high fistulae. According to our promising results for the cutting-seton technique, the proper use of this technique with respect to disadvantages can really be a helpful operation with good outcomes even for faecal incontinence. It should, therefore, be further evaluated or revised in a prospective study.

### Limitations

There are several weaknesses in our study. It is a retrospective study and therefore conclusions must be taken with caution. The number of patients is limited, and the study compares multiple procedures, thus making the number within each subset even smaller, particularly the subgroups undergoing anal fistula plugs or cutting-setons, which are essentially smaller than the other two subgroups. This can be partially explained by the fact that cutting-seton is considered an obsolete technique and thus, was not routinely performed. Anal fistula plug was a fairly new technique at the time of the treatment of the patients and was performed mostly by expert colorectal surgeons. There was not a single consensus according to the decision-making algorithm. Some operations were performed by residents and some by consultants with or without specialization in Proctology. Furthermore, concerning faecal incontinence, there is no information about the continence of the patients prior to the treatment of the anal fistula and there is no pre- or postoperative imaging for sphincter muscle assessment using weighted instruments such as anorectal manometry, endoanal ultrasonography or magnetic resonance imaging. The postoperative evaluation of faecal incontinence was either made during the clinical follow up or through the telephone-interview. There was no written questionnaire neither for faecal incontinence nor for quality of life.

## Methods

Between January 2005 and May 2013, we retrospectively analyzed all patients who underwent surgical treatment for anal fistula at the surgical department of the University Hospital of Basel. All patients with a cryptoglandular fistula were included. Exclusion criteria were the presence of chronic inflammatory bowel disease as well as previous operations for anal carcinoma or rectal cancer.

In the time period between January 2005 and May 2013, a surgical treatment for anal fistula was performed in 67 patients that met the criteria of our study. Two patients were excluded due to loss of follow-up. A total of 93 operations was performed in 79 fistulae. Surgeons had no guidelines to follow on how to perform the operation and each surgeon took the decision about the type of the technique that was going to be performed after the diagnostic proctoscopy and intraoperative assessment of the situs. The performed operations were fistulotomy, mucosal advancement flap, anal fistula plug and cutting-seton placement.

The primary endpoints were long-term closure rate, recurrence rate and re-operation rate in association to each surgical technique after 2 years. Secondary endpoints were persistent pain after 2 years, postoperative complications according to the Clavien-Dindo classification of surgical complications, postoperative wound infections and postoperative continence for gas, liquid and formed stool according to the Wexner Score^35^. Wexner score ranges from 0 to 20, where higher scores indicate a higher level of incontinence.

Closure rate was defined as a healed local situation with no fistula-related symptoms and no remaining wound after 2 years following each operation. Therefore, if a patient had a surgical treatment for a recurrence before 2 years after the initial operation, that patient was followed up for another 2 years. The last recurrence of all included patients was observed in September 2014 which was successfully treated and followed up accordingly. Recurrence rate was either defined as persisting fistula requiring further surgery, or as a new fistula in the same region during the follow-up. Fistula in a different region of the anal ring wasn’t counted as a recurrence. Therefore, if after surgical treatment of a fistula at 1 o’clock, a new fistula was diagnosed at 5 or 7 o’clock during follow up examination with no evident communication between the 2 compartments, there was no recurrence documented, and a new fistula was recorded.

For data collection, a database was established. The patients’ medical records were reviewed by the author under the supervision of the principal investigator and member of the ESCP (European Society of Coloproctology). All electronic and written medical data about inpatient information, information from outpatient follow up consultations and follow up phone calls were included in the database. When existing, information from other hospital stays and family doctor consultations were further included. The data collection and usage were carried out in accordance with the Declaration of Helsinki.

The study was approved by the Association of Swiss Ethics human subject Committee of northwest / central Switzerland (Project ID: 2018-02046) and all methods were performed in accordance with the relevant guidelines and regulations of this Committee. The need for obtaining informed patient consent for this retrospective analysis of anonymised patient data was waived by the Ethics Committee who approved the study protocols.

## Data Availability

The datasets generated during and/or analysed during the current study are available from the corresponding author on reasonable request.
